# Circulating Levels of Anti-C1q and Anti-Factor H Autoantibodies and Their Targets in Normal Pregnancy and Preeclampsia

**DOI:** 10.3389/fimmu.2022.842451

**Published:** 2022-03-31

**Authors:** Douwe Jan Dijkstra, A. Inkeri Lokki, Lobke Marijn Gierman, Nicole Veronique Borggreven, Carin van der Keur, Michael Eikmans, Kyra Andrea Gelderman, Hannele Laivuori, The FINNPEC Core Investigator Group, Hannele Laivuori, Ann-Charlotte Iversen, Marie-Louise P. van der Hoorn, Leendert Adrianus Trouw

**Affiliations:** ^1^ Department of Immunology, Leiden University Medical Center, Leiden, Netherlands; ^2^ Department of Bacteriology and Immunology, University of Helsinki and Helsinki University Hospital, Helsinki, Finland; ^3^ Medical and Clinical Genetics, University of Helsinki and Helsinki University Hospital, Helsinki, Finland; ^4^ Centre of Molecular Inflammation Research, Department of Clinical and Molecular Medicine, Norwegian University of Science and Technology, Trondheim, Norway; ^5^ Department of Immunopathology and Haemostasis, Sanquin Diagnostic Services, Amsterdam, Netherlands; ^6^ Institute for Molecular Medicine Finland, Helsinki Institute of Life Science, University of Helsinki, Helsinki, Finland; ^7^ Department of Obstetrics and Gynecology, Tampere University Hospital and Tampere University, Faculty of Medicine and Health Technology, Tampere Center for Child, Adolescent, and Maternal Health Research, Tampere, Finland; ^8^ Department of Obstetrics and Gynecology, Leiden University Medical Center, Leiden, Netherlands

**Keywords:** C1q, complement, factor H, autoantibodies, preeclampsia, pregnancy

## Abstract

Preeclampsia (PE) generally manifests in the second half of pregnancy with hypertension and proteinuria. The understanding of the origin and mechanism behind PE is incomplete, although there is clearly an immune component to this disorder. The placenta constitutes a complicated immune interface between fetal and maternal cells, where regulation and tolerance are key. Stress factors from placental dysfunction in PE are released to the maternal circulation evoking the maternal response. Several complement factors play a role within this intricate landscape, including C1q in vascular remodeling and Factor H (FH) as the key regulator of alternative pathway complement activation. We hypothesize that decreased levels of C1q or FH, or disturbance of their function by autoantibodies, may be associated with PE. Autoantibodies against C1q and FH and the concentrations of C1q and FH were measured by ELISA in maternal sera from women with preeclamptic and normal pregnancies. Samples originated from cohorts collected in the Netherlands (n=63 PE; n=174 control pregnancies, n=51 nonpregnant), Finland (n=181 PE; n=63 control pregnancies) and Norway (n=59 PE; n=27 control pregnancies). Serum C1q and FH concentrations were higher in control pregnancy than in nonpregnant women. No significant differences were observed for serum C1q between preeclamptic and control pregnancy in any of the three cohorts. Serum levels of FH were lower in preeclamptic pregnancies compared to control pregnancies in two of the cohorts, this effect was driven by the early onset PE cases. Neither anti-C1q autoantibodies nor anti-FH autoantibodies levels differed between women with PE and normal pregnancies. In conclusion, levels of anti-C1q and anti-FH autoantibodies are not increased in PE. C1q and FH are increased in pregnancy, but importantly, a decrease in FH concentration is associated with PE.

## Introduction

Preeclampsia (PE) is a vascular complication presenting in the second half of pregnancy. PE occurs in about 3% of pregnancies and is an important cause of both fetal and maternal morbidity (1). The disease is characterized by newly developed hypertension and proteinuria, or new-onset PE-associated signs in the absence of proteinuria, and can progress systemically to include organ dysfunction. Onset of PE is unpredictable, with the only cure being delivery of the placenta (2). Although the mechanism behind the development of PE is incompletely understood, its origin may involve improper placentation with incomplete remodeling of the uterine spiral arteries and development of a dysfunctional placenta with vascular malperfusion (3). As the pregnancy progresses and the fetus demands a fully functional placenta, the placenta releases increasing amounts of stress signals to the maternal circulation and the mother may eventually develop endothelial dysfunction and the clinical manifestation of PE.

In the context of healthy pregnancy, a well-functioning and regulated complement system is key. The complement system is an important component of the innate immune system, consisting of more than 30 protein factors that form three converging activation pathways (classical, lectin and alternative pathways) with their accessory regulators (4). Although classically known in the defense against infections, the complement system is also involved in clearance of immune complexes and dying cells, tissue regeneration and angiogenesis (5). Additionally, complement forms a bridge towards cellular immune responses, attracting and activating immune cells locally with activation fragments that act as anaphylatoxins and opsonins.

The initiator of the classical pathway of the complement system, C1q, binds directly to apoptotic cells and debris that results from tissue remodeling, labeling it for clearance (6). The relevance of C1q for a healthy pregnancy was highlighted by the observation of impaired labyrinth development and vessel remodeling in pregnant C1q-deficient mice compared to wildtype mice (7). Additionally, C1q-deficient mice are predisposed to present with PE-like symptoms and higher fetal loss (8). In human pregnancy, C1q is found throughout the maternal side of the placenta and is also locally produced by invading trophoblasts, the fetal cell type directly interacting with maternal cells at the maternal-fetal interface (7). In healthy pregnancy the local presence of C1q in the placenta does not result in overt complement activation (9). Human C1q deficiency is very rare and although it is studied in great detail because of its association with development of Systemic Lupus Erythematosus (SLE) and infections (10), no information is currently available on pregnancy outcomes.

Factor H (FH) is a key regulator of the alternative pathway because of its ability to block C3 convertase formation and its function as a cofactor for Factor I. In this way, FH plays an important role in the steady state protection of host cells throughout the body, especially circulating cells and endothelial cells. In addition, FH provides protection against excessive complement activation on apoptotic cells, which downregulate membrane-bound complement inhibitors (11). FH is abundantly present in the placenta, but the observed irregular distribution of FH in the PE placenta may suggest a disturbed balance between complement activation and regulation in PE (12). FH was even observed intracellularly in syncytiotrophoblast, although this FH may actually be of fetal rather than maternal origin (13).

Autoantibodies may target multiple complement proteins, including C1q and FH (14). Anti-C1q autoantibodies are typically present in diseases like SLE, Hypocomplementemic Urticarial Vasculitis Syndrome and Rheumatoid Vasculitis, but are also present in a fraction of healthy individuals (15). Importantly, anti-C1q autoantibodies do not deplete circulating C1q, but may amplify classical pathway driven complement activation (16) and dysregulate other processes driven by C1q (17). Autoantibodies to FH have been described in atypical Hemolytic Uremic Syndrome and C3-glomerulopathy as well as in antiphospholipid syndrome and other autoimmune diseases (18, 19). Anti-FH autoantibodies may have different functional consequences, as some form immune complexes leading to (partial) FH depletion, while other anti-FH autoantibodies affect the functional properties of FH (19). Consequences of these autoantibodies are often associated with autoimmune diseases, where they may drive or enhance pathogenesis. Several previous studies showed a relationship between anti-C1q autoantibodies and negative pregnancy outcomes in miscarriage, ectopic pregnancy and autoimmune thyroid disorders, but not for PE (20–22). A study focusing on pregnancy in lupus nephritis patients found that anti-C1q was not a predictor of PE (23).

We hypothesize that a balance between complement activation, needed for tissue remodeling and clearance of apoptotic cells in the placenta, and regulation is critical for a healthy pregnancy. This balance may be disturbed in PE, which could contribute to the underlying pathogenesis. Autoantibodies against complement factors may further hamper their proper function or incite unwanted immune activation. In this study, we therefore compared the serum levels of C1q and FH, and autoantibodies against these factors, in women with healthy or preeclamptic pregnancies.

## Materials and Methods

### Patients and Samples

Serum samples of women with preeclamptic and control pregnancies were derived from three cohorts collected in the Netherlands, Finland and Norway. PE was defined as hypertension (systolic blood pressure ≥140 mmHg or diastolic blood pressure ≥90 mmHg) and proteinuria (≥0.3 g/24h or ≥0.3 g/L), or newly developed PE-associated signs in the absence of proteinuria, with onset beyond 20 weeks of gestation. Women with normal pregnancies and no previous history of PE were included as controls.

The cohort from the Netherlands was collected at the Leiden University Medical Center (LUMC) and comprised 63 women with PE and 174 women with control pregnancies. Serum samples from these women were collected on the day of delivery, although it varied whether this was performed before or after the actual delivery. Additionally, a group of 51 nonpregnant women with a similar age profile as the pregnancy cohorts (median age 31 years, range 20-46), was included.

The cohort from Finland comprises samples of 181 preeclamptic and 63 control pregnancies from the Finnish Genetics of Pre-eclampsia Consortium (FINNPEC) that has been described before (24). In this cohort proteinuria could additionally be diagnosed based on two ≥1 readings on a dipstick in a random urine determination with no evidence of a urinary tract infection. Samples were collected at recruitment, 74% were taken prior to the day of delivery (range 1 to 31 days before delivery), while 25% of samples were taken on the day of delivery, one sample in the control pregnancy group was taken on the second day after delivery.

The Norwegian cohort was collected at St. Olavs and Haukeland University Hospitals and comprises sera from 59 women with PE and 27 women with control pregnancies. All samples in this cohort were collected before delivery on the day of delivery by caesarean section, without signs of being in labor. Caesarean sections for the control group were indicated due to breech position, suspected birth defects, previous obstetric history, or birth anxiety. Serum samples from Finland and Norway were stored frozen and were transported to LUMC for analysis. Ethical approval for the three cohorts was obtained at the individual centers (the Netherlands: P08.229/228, Finland: 149/EO/2007, Norway: REC 2012/1040).

### Anti-C1q Antibody ELISA

Antibodies against C1q were measured by QUANTA Lite Anti-C1q ELISA (Inova Diagnostics) according to the manufacturer’s protocol. Briefly, 100 µl of 1:101 diluted sera in Samples Diluent were incubated in ELISA plate wells for 30 minutes at room temperature. Wells were washed 3 times with wash buffer, incubated with 100 µl horseradish peroxidase (HRP) conjugated anti-IgG and incubated for 30 minutes at room temperature. After washing 3 times, wells were incubated with 100 µl TMB Chromogen for 30 minutes at room temperature, followed by addition of 100 µl acidic HRP Stop Solution. Absorbance values were read at 450 nm and used to calculate anti-C1q units based positive control samples included in the kit. The cut-off for positivity was 20 units, as recommended by the manufacturer.

### Anti-FH Antibody ELISA

Measurement of antibodies against FH was performed using an in-house developed ELISA. Nunc MaxiSorp ELISA plates (ThermoScientific) were coated with 50 µl of 10 µg/ml FH (Complement Technology) in 0.1M bicarbonate coating buffer (pH 9.6) and incubated overnight at 4°C. Wells were washed 3 times with PBS/0.05% Tween, blocked with 100 µl PBS/1% BSA for 1 hour at 37°C and washed 3 times. Serum samples were diluted 1:50 in PBS/0.05% Tween/1% BSA (PTB) and 50 µl sample was incubated in wells for 1 hour at 37°C. After washing 3 times, 50 µl 0.11 µg/ml Goat anti-human IgG-biotin (Invitrogen) was added, followed by incubation for 1 hour at 37°C and washing 3 times. Secondary detection consisted of 50 µl 0.5 µg/ml streptavidin-HRP (ThermoScientific), incubated for 1 hour at 37°C. After the final washing sequence, 50 µl 2,2’-azino-bis(3-ethylbenzothiazoline-6-sulfonic acid (ABTS)/0.015% H_2_O_2_ was added. Absorbance was read at 415 nm. Anti-FH units (U) were determined based on standard curve of anti-FH standard (180kU; Sanquin Diagnostics, Amsterdam, the Netherlands) by an Excel-based logit calculation. Cut-off for positivity was set at 95^th^ percentile of all control pregnancy samples combined.

### C1q ELISA

To measure the concentration of C1q in serum samples, an in-house ELISA was performed as described before (25). Nunc MaxiSorp ELISA plates (ThermoScientific) were coated with 50 µl 2.5 µg/ml mouse anti-human C1q monoclonal antibody (mAb) 2204 (kind gift Prof. C. van Kooten, Dept Nephrology, LUMC) in 0.1M bicarbonate coating buffer (pH 9.6) and incubated overnight at room temperature. Wells were washed 3 times with PBS/0.05% Tween and blocked with 100 µl PBS/1% BSA and incubated for 1 hour at 37°C. After washing, 50 µl of serially diluted serum samples in PTB, as well as a standard curve from a pool of normal human serum were added to the wells, followed by incubation for 1 hour at 37°C and washing 3 times. Wells were then incubated with 50 µl 1:2000 diluted rabbit anti-human C1q (DAKO) for 1 hour at 37°C. After washing, 50 µl 1:2000 goat anti-rabbit-HRP (DAKO) was added for detection and wells were incubated for 1 hour at 37°C. Following the final washing sequence, wells were incubated with 50 µl ABTS/0.015% H_2_O_2_ and absorbance was read at 415 nm. C1q concentrations were calculated based on the standard curve of a reference serum.

### FH ELISA

Concentration of FH in serum was determined by an in-house ELISA on Nunc MaxiSorp ELISA plates (ThermoScientific). Wells were coated with 50 µl 0.5 µg/ml mouse anti-FH (clone FH.16; Sanquin) in bicarbonate coating buffer (pH 9.6) and incubated overnight at room temperature. After washing 3 times with PBS/0.05% Tween, wells were blocked with 100 µl PBS/1% BSA for 1 hour at 37°C. Samples were diluted 1:2000 and 1:4000 in PTB, and 50 µl was added to the wells. For calculation of FH concentration, a standard curve of a reference serum with known FH concentration was added on each plate. Wells were incubated for 1 hour at 37°C and washed, then incubated with 50 µl 0.25 µg/ml biotinylated mouse anti-human FH mAb OX-23 for 1 hour at 37°C. After washing, detection was performed by incubating the wells with 50 µl 0.5 µg/ml streptavidin-HRP (ThermoScientific) for 1 hour at 37°C. Following the final wash, wells were stained with 50 µl ABTS/0.015% H_2_O_2_ and absorbance was read at 415 nm. FH concentrations were calculated based on the standard curve of a reference serum.

### Statistical Analysis

All statistical analyses were performed using GraphPad Prism 8 for Windows (GraphPad Software, San Diego, CA, USA). Normal distribution of data was examined by D’Agostino & Pearson normality test. Comparisons of numerical data were performed by Mann-Whitney U test, whereas categorical data was analyzed with Fisher’s exact test. Analysis of samples matched for gestational age (in the cohort from Finland) was performed by forming pairs with maximum 2 days difference in gestational age, C1q and Factor H concentrations were then compared between pairs by paired t test. Correlation between data was analyzed by Spearman’s rank correlation.

## Results

### Patient Characteristics

To study the relationship between the presence of anti-complement autoantibodies and complement protein levels and PE we analyzed serum samples obtained from cohorts from the Netherlands, Finland and Norway, comprising a total of 289 preeclamptic pregnancies and 264 control pregnancies. Three cohorts from different countries were investigated to provide a robust basis for conclusions. Overall, the three cohorts displayed the expected clinical characteristics associated with PE, such as increased diastolic blood pressure, proteinuria and lower gestational age in the PE-complicated pregnancies as compared to the control pregnancies (**Table 1**). Both gravidity and parity were in general lower in the PE group than in controls although some differences existed between the cohorts. The proportion of deliveries performed through Caesarean section was not significantly different between the groups in any of the cohorts. Maternal body mass index (BMI) was higher in the Finnish PE group as compared to the control group, no difference in BMI was present in Norway, while BMI data from the Netherlands was not available. When comparing the three cohorts of PE cases, the cohort from Finland stands out with a higher median gestational age and fetal birthweight, (**Table 2**). Moreover, among both PE cases and controls, a lower frequency of Caesarean sections is present in the Finnish cohort.

**Table 1:** Clinical characteristics of the included subjects.

The Netherlands	Preeclampsia (n = 63)	Control (n = 174)	p-value
Maternal age (years)	31 (18-46)	33 (23-42)	**0.0117** ^#^
Gestational age (days)	220 (170-283)	275 (266-297)	**<0.0001** ^#^
Fetal birthweight (g)	1246 (380-4030)	3598 (2445-5100)	**<0.0001** ^#^
Blood pressure (mmHg)	104 (90-160)	75 (55-90)	**<0.0001** ^#^
Proteinuria (mg/24hr)	2300 (310-14000) ^(16)^	N/A	N/A
Gravidity	2 (1-9)	3 (1-9)	**<0.0001** ^#^
Parity	0 (0-5)	1 (0-6)	**<0.0001** ^#^
Caesarean section (%)	49 (78%)	113 (65%)	0.0814^$^
**Finland**	**Preeclampsia (n = 181)**	**Control (n = 63)**	**p-value**
Maternal age (years)	32 (18-47)	31 (21-43)	**<0.0001** ^#^
Gestational age (days)	246 (165-286) ^(1)^	280 (167-297) ^(1)^	**<0.0001** ^#^
Fetal birthweight (g)	2775 (310-5110)	3470 (330-4748)	**<0.0001** ^#^
Blood pressure (mmHg)	111 (91-173)	84 (67-130) ^(1)^	**<0.0001** ^#^
Proteinuria (mg/L)	3200 (300-19100) ^(3)^	200-270 ^(60)^	**<0.0001** ^#^
Gravidity	1 (1-18)	2 (1-5) ^(1)^	**<0.0001** ^#^
Parity	0 (0-12)	1 (1-2) ^(1)^	0.4621^#^
Caesarean section (%)	90 (50%)	25 (40%) ^(1)^	0.2389^$^
BMI (start of pregnancy)	23.90 (18.20-48.40)	22.90 (18.20-39.10) ^(1)^	**<0.0001** ^#^
**Norway**	**Preeclampsia (n = 45)**	**Control (n = 27)**	**p-value**
Maternal age (years)	30 (20-45)	33 (23-41)	**0.0447** ^#^
Gestational age (days)	223 (178-275)	274 (265-294)	**<0.0001** ^#^
Fetal birthweight (g)	1286 (550-5010)	3510 (2800-4330)	**<0.0001** ^#^
Blood pressure (mmHg)	100 (80-130)	75 (60-99)	**<0.0001** ^#^
Proteinuria (Yes/No)	Yes: 45	No: 26 ^(1)^	**<0.0001** ^$^
Gravidity	2 (1-13)	2 (1-5)	0.0743^#^
Parity	0 (0-3)	1 (0-3)	**0.0095** ^#^
Caesarean section (%)	45 (100%)	27 (100%)	>0.9999^$^
BMI (start of pregnancy)	24.4 (18.2-38.4) ^(4)^	24.6 (16.4-33.8) ^(2)^	0.522

For patient characteristics, median and range are indicated. For blood pressure, the highest diastolic blood pressure is used. Where information was not available for all subjects, the number of missing data points is indicated in superscript. N/A, not applicable. Statistical significance calculated by Mann Whitney U test (#) or Fisher’s exact test ($). P-values <0.05 are indicated in bold.

**Table 2:** Comparison of clinical characteristics between the three independent cohorts in control pregnancies and in PE cases.

	Control pregnancies			
Parameter	The Netherlands (n=174)	Finland (n=63)	Norway (n=27)	p-value
Maternal age (years)	33 (23-42)	31 (21-43)	33 (23-41)	0.032
Gestational age (days)	275 (266-297)	280 (167-297)^(1)^	274 (265-294)	0.205
Fetal birthweight (g)	3598 (2445-5100)	3470 (330-4748)	3510 (2800-4330)	0.089
Blood pressure (mmHg)	75 (55-90)	84 (67-130)^(1)^	75 (60-99)	**<0.0001**
Gravidity	3 (1-9)	2 (1-5)^(1)^	2 (1-5)	**0.0006**
Parity	1 (0-6)	1 (1-2)^(1)^	1 (0-3)	**<0.0001**
Caesarean section (%)	113 (65%)	25 (40%)^(1)^	27 (100%)	**<0.0001**
	**Preeclampsia**			
**Parameter**	**The Netherlands (n = 63)**	**Finland (n = 181)**	**Norway (n = 45)**	**p-value**
Maternal age (years)	31 (18-46)	32 (18-47)	30 (20-45)	0.409
Gestational age (days)	220 (170-283)	246 (165-286)^(1)^	223 (178-275)	**<0.0001**
Fetal birthweight (g)	1246 (380-4030)	2775 (310-5110)	1286 (550-5010)	**<0.0001**
Blood pressure (mmHg)	104 (90-160)	111 (91-173)	100 (80-130)	**<0.0001**
Gravidity	2 (1-9)	1 (1-18)	2 (1-13)	0.532
Parity	0 (0-5)	0 (0-12)	0 (0-3)	0.038
Caesarean section (%)	49 (78%)	90 (50%)	45 (100%)	**<0.0001**

Where information was not available for all subjects, the number of missing data points is indicated in superscript. All comparisons were performed with Kruskal-Wallis test, except for the mode of delivery, which was analyzed by Chi-square test. P-values <0.05 are indicated in bold.

### Anti-C1q Autoantibodies and C1q

**Figure 1:**
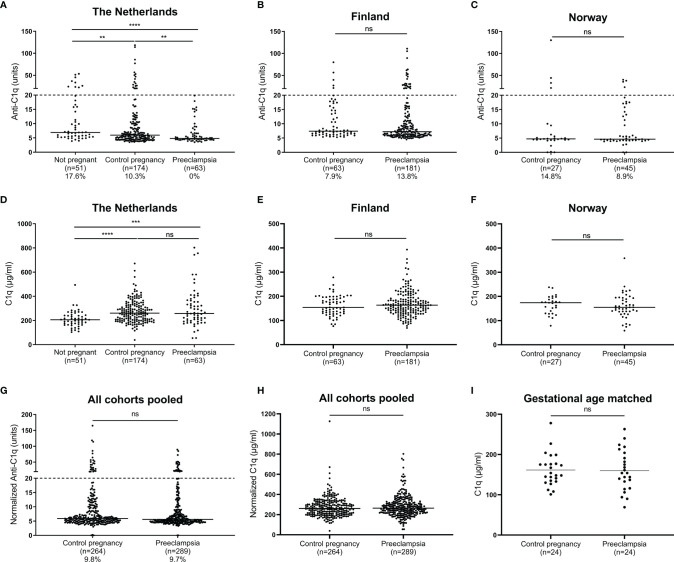
Anti-C1q antibodies and C1q in healthy or PE pregnancy. Anti-C1q antibodies measured in maternal serum in cohorts from the Netherlands **(A)**, Finland **(B)** and Norway **(C)**, with percentages under the graph indicating the proportion samples deemed anti-C1q positive (>20 units, also indicated by dashed line). C1q concentration measured in maternal serum in cohorts from the Netherlands **(D)**, Finland **(E)** and Norway **(F)**. Data from different cohorts was normalized and pooled for anti-C1q **(G)** and C1q **(H)**. For the cohort from Finland, a subanalysis with samples matched for gestational age was additionally performed **(I)**. Comparisons tested by Mann-Whitney, or paired t test for panel I; ns, not significant (p > 0.05); **, 0.001 < p < 0.01; ***, 0.0001 < p < 0.001; ****, p < 0.0001.

Anti-C1q antibodies were absent in maternal serum of preeclamptic pregnancies in the cohort from the Netherlands, while 10.3% of control pregnancy sera and 17.6% of sera from nonpregnant women were positive for anti-C1q (**Figure 1A**). However, no significant differences between control and preeclamptic pregnancies were observed for anti-C1q levels in the other cohorts (**Figures 1B, C, G**).

For serum C1q concentration, no significant differences were observed between the PE and control pregnancies in any of the cohorts (**Figures 1D–F**). Analysis of pooled data from all cohorts and gestational age-matched samples confirmed the lack of association between C1q concentration and PE (**Figures 1H, I**). Interestingly, the serum C1q concentration was higher for both control and preeclamptic pregnancies than for nonpregnant women (**Figure 1D**).

### Anti-FH Autoantibodies and FH

**Figure 2:**
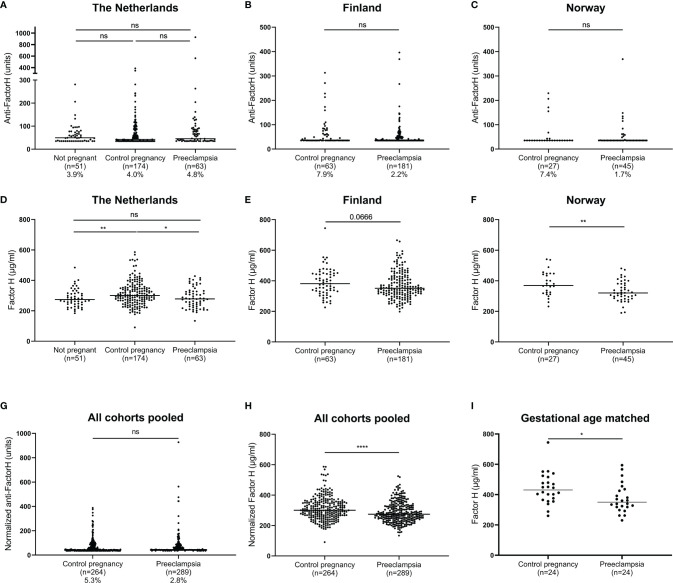
Anti-Factor H antibodies and Factor H in healthy or PE pregnancy. Anti-Factor H antibodies measured in maternal serum in cohorts from the Netherlands **(A)**, Finland **(B)** and Norway **(C)**, dotted line indicates the detection limit at 35.2 units. Factor H concentration measured in maternal serum in cohorts from the Netherlands **(D)**, Finland **(E)** and Norway **(F)**. Data from different cohorts was normalized and pooled for anti-FH **(G)** and FH **(H)**. For the cohort from Finland, a subanalysis with samples matched for gestational age was additionally performed **(I)**. Comparisons tested by Mann-Whitney, or paired t test for panel I; ns, not significant (p > 0.05); *, 0.05 < p < 0.01; **, 0.001 < p < 0.01; ****, p < 0.0001.

We tested whether dysregulation of FH by the presence of autoantibodies is associated with PE, but no significant differences were observed regarding the levels of anti-FH autoantibodies between PE and control pregnancies (**Figures 2A–C, G**). However, importantly we observed significantly decreased levels of FH comparing the preeclamptic pregnancies to healthy pregnancies. In the cohorts from the Netherlands and Norway, the FH concentration was significantly lower in the PE group compared to controls pregnancies (**Figures 2D, F**). In samples from Finland, a similar trend of lower FH concentration in the PE group was observed (p=0.067) (**Figure 2E**). When pooled data from all cohorts were analyzed, the lower FH concentration remained significant (**Figure 2H**). Additionally, an analysis of samples matched for gestational age in the cohort from Finland shows the same result, excluding the possibility of data skewing by lower gestational ages in PE (**Figure 2I**). Similar to what was observed for C1q, women with control pregnancies showed higher FH concentration than nonpregnant controls (**Figure 2D**).

### Study of the Association of Complement and Autoantibodies With Clinical Parameters and Subgroups of PE Patients

To further investigate the relationship between PE and C1q, FH or autoantibodies targeting these proteins, Spearman correlations with clinical parameters associated with PE were explored for all PE patients (**Table 3**). The clinical parameters included proteinuria and diastolic blood pressure, fetal birthweight and gestational age at delivery, (**Table 1**). Interestingly, a positive correlation was found between the mother’s BMI and serum FH concentration in the cohorts from Finland and Norway (Spearman r=0.288; p=8.6e-5 and r=0.351; p=0.024 respectively). However, no other consistent significant correlations were found relating to PE symptoms or other relevant clinical parameters.

**Table 3:** Correlation between clinical parameters and C1q, FH and autoantibodies in preeclamptic pregnancies.

	The Netherlands (n = 63)							
Parameter	Anti-C1q		C1q		Anti-FH		FH	
	Spearman r	p-value	Spearman r	p-value	Spearman r	p-value	Spearman r	p-value
Gestational age	-0.199	0.118	0.050	0.696	-0.248	0.050	0.023	0.861
Proteinuria	-0.243	0.100	-0.200	0.178	0.146	0.326	0.090	0.549
Highest diastole	0.078	0.541	-0.088	0.495	0.179	0.160	0.101	0.430
Fetal birthweight	-0.150	0.241	-0.074	0.566	**-0.291**	**0.021**	0.031	0.806
	**Finland (n = 181)**							
	**Anti-C1q**		**C1q**		**Anti-FH**		**FH**	
	**Spearman r**	**p-value**	**Spearman r**	**p-value**	**Spearman r**	**p-value**	**Spearman r**	**p-value**
Gestational age	-0.044	0.562	0.026	0.734	0.082	0.271	0.090	0.227
Proteinuria	-0.017	0.826	-0.069	0.360	0.008	0.911	**-0.157**	**0.037**
Highest diastole	-0.145	0.051	-0.073	0.330	**-0.198**	**0.007**	0.100	0.180
Fetal birthweight	-0.117	0.118	-0.023	0.756	0.005	0.948	0.135	0.071
BMI	-0.111	0.135	0.004	0.958	0.034	0.649	**0.288**	**8.57e-5**
	**Norway (n = 45)**							
	**Anti-C1q**		**C1q**		**Anti-FH**		**FH**	
	**Spearman r**	**p-value**	**Spearman r**	**p-value**	**Spearman r**	**p-value**	**Spearman r**	**p-value**
Gestational age	0.016	0.920	-0.125	0.412	0.042	0.785	**0.327**	**0.029**
Highest diastole	0.006	0.967	-0.155	0.311	-0.194	0.203	0.071	0.644
Fetal birthweight	0.061	0.691	-0.257	0.089	-0.025	0.873	0.250	0.098
BMI	0.025	0.877	0.306	0.052	0.008	0.961	**0.351**	**0.024**

P-values <0.05 are indicated in bold.

Further analyses were performed on subgroups of PE patients based on time of disease onset or presence of fetal growth restriction (FGR) (**Table 4**). Early onset of PE (gestational age below 34 weeks) was associated with decreased FH concentration compared to late onset, this difference was significant in cohorts from Finland and Norway, while a similar trend was observed in the cohort from the Netherlands. PE with FGR was associated to higher serum levels of anti-FH antibodies compared to preeclampsia with normal fetal growth, but this difference was only statistically significant in the cohort from the Netherlands.

**Table 4:** Analysis of complement factors C1q, FH and autoantibodies in subgroups based on early onset of disease or presence of fetal growth restriction.

	The Netherlands		Finland		Norway	
	Early (n=50) vs late (n=13) onset PE	p-value	Early (n=36) vs late (n=145) onset PE	p-value	Early (n=33) vs late (n=12) onset PE	p-value
Anti-C1q	4.75 – 5.00	0.407	7.95 – 7.10	0.198	4.60 – 4.90	0.854
C1q	265 – 225	0.753	165 – 162.6	0.881	155 – 150	0.608
Anti-FH	56.7 – 37.3	0.122	35.2 – 35.2	0.194	35.2 – 35.2	0.392
FH	276 – 306	0.537	**322.5 – 354**	**0.019**	**306 – 381**	**0.001**
	**The Netherlands**		**Finland**		**Norway**	
	**PE with (n = 17) vs without (n = 46) FGR**	**p-value**	**PE with (n = 84) vs without (n = 97) FGR**	**p-value**	**PE with (n = 33) vs without (n = 12) FGR**	**p-value**
Anti-C1q	4.60 – 4.95	0.715	7.40 – 7.10	0.294	4.50 – 5.70	0.391
C1q	257 – 256	0.772	166.8 – 155.9	0.119	159 – 146	0.377
Anti-FH	**79.0 – 41.9**	**0.029**	35.2 – 35.2	0.369	35.2 – 35.2	0.253
FH	282 – 277	0.936	344.5 – 354	0.222	318 – 363	0.255

Medians of each groups are indicated, together with significance as determined with Mann-Whitney test. Early onset was defined as gestational age at delivery below 34 weeks; fetal growth restriction (FGR) was defined as gestational age-adjusted birthweight up to percentile 10 (of growth curve for respective country). P-values <0.05 are indicated in bold.

## Discussion

In the current study, we investigated the relation between the pregnancy complication PE and the circulating levels of complement factors C1q and FH, as well as autoantibodies against these factors. We observed that the presence of autoantibodies against C1q or FH is not associated with PE. Circulating C1q and FH levels were higher in pregnant than in nonpregnant women. Decreased serum concentrations of FH were associated with PE, while this was not the case for serum C1q concentrations. Serum FH levels were lower in the subset of PE cases with earlier onset, compared to late onset cases.

Although the etiology of PE remains unclear, multiple immune mechanisms have been proposed to play a role, as the placenta constitutes a unique immune environment. Differentially expressed levels of fetal Human Leukocyte Antigen (HLA), especially HLA-G (26–28), immune cell influx (29) and cytokine expression (30) have been reported in preeclamptic placentas. Complement has also been implicated in the development of PE. Our data now add to this understanding that the circulating levels of the endogenous complement inhibitor FH are decreased in PE. Several studies linked anti-C1q autoantibodies to various negative pregnancy outcomes, but not to PE (20–23). The current study reinforces that the presence of anti-C1q does not associate with PE. Likewise, no evidence for a link between anti-FH and PE was observed. These results contradict the hypothesis of a contribution by complement dysregulation by autoantibodies to development of PE.

C1q has multiple functions during pregnancy, e.g. in angiogenesis, tissue remodeling and clearance of cellular debris. These functions are supported by the ability of C1q to recognize exposed or altered self-structures, facilitating clearance without overt inflammation (31). Next to observations in human pregnancy, data from experiments in mice indicate a key role for C1q in placentation as the litter size in C1q deficient mice is smaller as compared to wild-type mice (7). The notion that C1q plays an important role in pregnancy fits with our finding of increased C1q levels in pregnant women, both complicated and control, as compared to nonpregnant women. Especially the role C1q plays in the clearance of apoptotic cells and debris is postulated to be important in the case of PE. Fragments of apoptotic syncytiotrophoblast from the placenta may enter the maternal circulation and the complement system assists in clearing these fragments by labelling them for phagocytosis. One previous study showed decreased C1q in severe PE cases (32), however this is contradicted by our finding that there was no difference in C1q levels between PE patients and controls.

In a prospective study increased FH and C1q levels were reported in maternal plasma of later PE patients relative to controls at 6 to 13 weeks of pregnancy (33). However, these differences were not observed in the second and third trimesters. The current study investigated only third trimester samples and will therefore have missed differences in complement protein levels in the first trimester. However, the current study does find a difference in third trimester serum FH level, which was not observed in the previous study by He and colleagues.

The balance between complement activation and regulation is key in a healthy pregnancy, which is substantiated by findings that FH levels increase during pregnancy (32, 34). This conclusion is corroborated by our results where women with control pregnancies had higher serum FH concentration than nonpregnant women. Abnormalities in activating and regulating components of the alternative complement pathway have been reported in PE. Multiple investigations found increased levels of activation fragment Bb in PE cases, indicating higher than normal alternative pathway activation (35, 36). Another study found increased Bb as well as decreased FH levels in PE patients, although this study was limited by relatively small samples size and a focus on only severe PE cases (32). Additionally, genetic variants in genes encoding the regulators Factor I and membrane cofactor protein, but not FH, were linked to PE (37). This trend of disbalance of the alternative pathway in PE is supported by our finding that FH concentration is decreased in PE patients. As the main fluid-phase complement regulator FH is conceivably of key importance in preventing or limiting C1q driven complement activation in the placenta. Aberrant FH levels or disturbed FH function would then result in the increased levels of complement activation as reported for PE.

Early onset PE cases showed lower FH concentration than late onset cases, implying that early onset cases were driving the lower FH concentration found in the general PE group compared to control pregnancies. This may also explain why FH concentration was not significantly lower in the cohort from Finland, as there were relatively fewer early onset cases in this cohort than in cohorts from the Netherlands and Norway. The larger variety in sampling timepoints in the Finnish cohort could also be involved. This study also investigated relations between the experimental data and relevant clinical parameters related to PE. No consistent significant correlations were found, indicating that within the PE pregnancies FH is not correlated with more severe disease. The observed correlation between BMI and FH is in line with earlier links found between FH and BMI (38, 39).

The strengths of the current analysis include the side-by-side comparison of three independent well-documented European cohorts, together comprising sizable numbers of cases and controls. In addition, all measurements were performed in the same lab with the same assays. Moreover, the current study also investigated correlations with several clinical parameters that could indicate severity of PE. The weaknesses of the study include the not completely identical sampling time points in the cohorts as samples in the Netherlands and Norway were collected on the day of delivery, while samples in Finland were mainly (74%) collected before the day of delivery, with an interval up to 31 days. Sample collection and processing may possibly have led to some *in vitro* complement activation. Importantly, such pre-analytical steps may impact on hemolytic activity of complement activity of samples or on complement activation fragments but does typically not impact on complement protein levels as measured by sandwich ELISA. If such effect may be present in a subset of the samples, then this is at least partially mitigated by the fact that samples for PE and controls were handled similarly. Furthermore, the focus on systemic levels in this investigation may not be fully representative of local effects in the placenta, leaving the question of cause and consequence in the etiology of PE open.

In conclusion, circulating levels of anti-FH and anti-C1q are not associated with the occurrence of PE. Circulating levels of C1q and FH are increased in healthy pregnancies as compared to nonpregnant controls. Importantly, circulating levels of FH are decreased in PE as compared to control pregnancy. Exactly how C1q is involved in the processes of placentation and pregnancy as a whole is still under investigation, but a key aspect appears to be that C1q is mediating its effect in the absence of clear complement activation (9). C1q deficiency is strongly associated with autoimmune disease SLE, as a result of insufficient cellular waste clearance (40). The increased C1q levels found during pregnancy could be a way to avoid accumulation of waste from placental remodeling. Upregulation of FH during healthy pregnancy could be a way to counteract excess complement activation, but FH has also been shown to promote a tolerogenic phenotype in dendritic cells, hinting at a noncanonical function for FH (41). Failure to increase FH level during pregnancy may therefore result in insufficient immune regulation contributing to development of PE. Further research will have to disclose what share of PE etiology can be ascribed to a disrupted equilibrium of complement activation and regulation.

## Data Availability Statement

The raw data supporting the conclusions of this article will be made available by the authors, without undue reservation.

## Ethics Statement

Ethical approval for the three cohorts was obtained locally, from the Medical Research Ethics Committee of Leiden University Medical Center, the Coordinating Ethics Committee, Hospital District of Helsinki and Uusimaa or the Regional Committee for Medical Research Ethics Central Norway, respectively. The patients/participants provided their written informed consent to participate in this study.

## The Finnish Genetics of Pre-Eclampsia Consortium (FINNPEC) Core Investigator Group

The FINNPEC core investigator group consists of the following persons:

Hannele Laivuori, principal investigator
Seppo Heinonen
Obstetrics and Gynecology, University of Helsinki and Helsinki University Hospital, Helsinki, Finland
Eero Kajantie
PEDEGO Research Unit, Medical Research Center Oulu, Oulu University Hospital and University of Oulu, Oulu, Finland
Public Health Promotion Unit, National Institute for Health and Welfare, Helsinki and Oulu, Finland
Children’s Hospital, University of Helsinki and Helsinki University Hospital, Helsinki, Finland
Department of Clinical and Molecular Medicine, Norwegian University of Health and Technology, Trondheim, Norway
Juha Kere
Department of Biosciences and Nutrition, Karolinska Institutet, Huddinge, Sweden
Folkhälsan Research Center and Stem Cells and Metabolism Research Program, University of Helsinki, Helsinki, Finland
Katja Kivinen
Institute for Molecular Medicine Finland, Helsinki Institute of Life Science, University of Helsinki, Helsinki, Finland
Anneli Pouta
Department of Government Services, National Institute for Health and Welfare, Helsinki, Finland

## Author Contributions

ME, M-LvdH, and LT designed the study. DD, NB, and CvdK performed the laboratory work. DD, AL, and LG analyzed the data. DD wrote the draft manuscript. All authors provided feedback to the manuscript and approved it.

## Funding

LT has received funding from the European Research Council (ERC) under the European Union’s Horizon 2020 research and innovation program (grant agreement No 724517).

## Conflict of Interest

The authors declare that the research was conducted in the absence of any commercial or financial relationships that could be construed as a potential conflict of interest.

## Publisher’s Note

All claims expressed in this article are solely those of the authors and do not necessarily represent those of their affiliated organizations, or those of the publisher, the editors and the reviewers. Any product that may be evaluated in this article, or claim that may be made by its manufacturer, is not guaranteed or endorsed by the publisher.
